# Traditional healing and mycetoma management in East Sennar State (Sudan): a qualitative exploration

**DOI:** 10.1186/s12939-025-02641-w

**Published:** 2025-10-29

**Authors:** Mohamed Elsheikh, Mei Trueba, Shahaduz Zaman

**Affiliations:** 1 Global Health and Infection Department, Brighton and Sussex Medical School, Brighton, UK; 2 Department of Physiology, Neuroscience and Behavioural Sciences, St Georges University, St George, Grenada

**Keywords:** Mycetoma, Traditional medicine, Health behavior, Medical anthropology, Healthcare system

## Abstract

**Background:**

Mycetoma is a neglected tropical disease with significant physical, social, and economic consequences. In Sudan, biomedical services dominate health policy and funding, often marginalising coexisting traditional healing systems. Understanding the interplay between these parallel systems is essential for improving patient outcomes.

**Objective:**

To explore the role of traditional healers in mycetoma management in Sudan, examine patients’ health-seeking behaviours, and analyse the power dynamics shaping healthcare pathways.

**Methods:**

We conducted a qualitative study using a critical medical anthropology framework. Data were collected through in-depth interviews, focus group discussions, and environmental observations, including analysis of health promotion materials. Participants included individuals with mycetoma, their families, carers, biomedical practitioners, and traditional healers. Data were thematically analysed to identify patterns in help-seeking, treatment experiences, and inter-system relationships.

**Results:**

Traditional healers were found to be the first point of contact for most people with mycetoma. Reliance on healers was influenced not only by economic barriers but also by cultural trust, social support, and systemic inequities. Biomedical practitioners frequently perceived healers as a cause of treatment delays, reflecting entrenched power asymmetries between health systems. Health promotion materials reinforced biomedical dominance and contributed to the marginalisation of traditional healing. Patients’ experiences were shaped by both interpersonal and institutional power relations.

**Conclusions:**

Traditional healers play a crucial role in sustaining access to care for people with mycetoma in Sudan. Effective collaboration between health systems will require addressing the structural and relational power imbalances that currently hinder integration. Recognising traditional healers as healthcare stakeholders is essential for culturally appropriate and equitable health interventions.

## Introduction

Notions of healing are central to all medical systems, encompassing a diverse array of practices embedded in cultural, social, and spiritual contexts. Anthropological research has long documented the wide variation in healing traditions across the globe, revealing both universal elements—such as defined healer–patient roles, ritualised interventions, and culturally meaningful metaphors—and culture-specific features shaped by local cosmologies and historical trajectories [1]. In many societies, traditional healing extends beyond the scope of codified “traditional medicine” to integrate ritual, community solidarity, and spiritual mediation, thereby situating illness and recovery within a broader framework of meaning and moral order [2].

In this paper we use the term “traditional healing” deliberately to highlight the interplay of material, social, and symbolic dimensions in care for mycetoma, and to situate these practices within the wider anthropological literature. The study of traditional healing can improve understanding of how different individuals shape knowledge of anatomical and physiological function, body image, and the meanings attached to symptoms. It describes how cultural socialisation practices differentially influence the health and illness behaviours of males and females, and recognises the beliefs and practices, values, knowledge, and traditions framed by the culture and worldview of a people [3]. Traditional healers have long been integral to healthcare in many communities, especially where conventional medical systems are inaccessible or culturally incompatible. In such contexts, the methods used by traditional healers, often holistic and deeply rooted in local beliefs, offer not only a therapeutic approach but also promote cultural continuity and personal empowerment [4]. By utilizing indigenous healthcare modalities, individuals can maintain a sense of autonomy over their health decisions, fostering higher community self-esteem and trust in care practices that reflect their values [5, 6]. Cooperative efforts between traditional and modern healthcare systems could significantly enhance health service delivery, particularly in underserved areas [7]. In such contexts, community, cultural, and personal self-esteem can be raised through people’s use of their own, instead of foreign, modalities of care to maintain or improve their health [8]. In this paper, we describe the knowledge and services provided by traditional healers towards mycetoma in a East Sennar State, Sudan, and the associated mycetoma management pathways. In so doing, we use insights from social sciences to ask whether and how traditional healing practices are, or should be, integrated into mainstream biomedicine.

In regions with scarce medical resources, with clashing transitions between modern and ancient health cultures, the approach to healthcare is often based on a person’s own understanding of their health, life, and being [9]. In 1978, at the International Conference on Primary Health Care (PHC) held at Alma Ata, the World Health Organization (WHO) recommended cooperation between traditional and Western, or so-called ‘modern’ – biomedical – systems of healthcare [8, 10]. Traditional medicine is very popular in most regions of sub-Saharan Africa. It is estimated that traditional healers cover 80–90 per cent of healthcare in Africa and that 80 per cent of the global population seek out traditional healers to meet their primary healthcare needs [11–13]. In 2020, Labhardt et al.. listed the most frequently mentioned reasons for seeking a traditional healer in Cameroon: consistency with local cultural values and beliefs; a therapeutic healer-patient relationship; proximity; and lower cost compared with biomedical healthcare facilities. In addition to being accessible and affordable, traditional medicine is often deeply embedded within, and aligned to, local or inherited belief systems, a connection that is particularly pronounced in rural settings. Given the significant number of people seeking care from traditional practitioners, numerous health programmes have attempted to involve traditional healers, especially within HIV⁄AIDS education and care packages [14].

Mycetoma is a chronic, granulomatous, devastating neglected disease of the skin that is common in tropical and subtropical regions. Mycetoma commonly affects young adults, particularly males aged between 20 and 40 years [15], and also mostly those within low- and middle-income countries. Associated with rural agricultural practices [16], people of low socio-economic status and manual workers, such as agriculturalists, labourers, and herdsmen, are the most commonly and worst affected [17]. Sudan remains the homeland of the disease, with the highest number of cases (10,608) globally being reported from Sudan [18]. Mycetoma affects subcutaneous tissues, often leading to severe deformities, disability, and even death if untreated. It is primarily caused by fungal organisms (eumycetoma) or bacteria (actinomycetoma) and is predominantly found in tropical and subtropical regions, particularly in areas with arid climates and low humidity, such as Sudan. Globally, the exact burden of mycetoma remains uncertain due to underreporting and limited epidemiological data [17]. In Sudan, mycetoma is prevalent in areas including Sennar, Al Jazirah, and Khartoum states, as environmental factors such as proximity to water bodies, soil composition, and vegetation, particularly the presence of Acacia trees, are linked to its occurrence [19]. A study conducted in Sudan’s Eastern Sennar locality found a prevalence rate of 0.87%, with most cases occurring in males between the ages of 31 and 45 [19]. The disease is often diagnosed late due to poor healthcare access, leading to extensive tissue damage and the need for surgical interventions, such as amputations, which further exacerbate the socio-economic impact on affected individuals and their families. Despite its severe consequences, mycetoma remains a neglected tropical disease, and there is an urgent need for better prevention, early detection, and integrated management strategies to alleviate its burden, particularly in endemic areas like Sudan [20].

Traditional medicine contributes significantly to the treatment of mycetoma in Sudan. According to Kunna et al., almost all of their study participants (389) had visited a traditional healer at some stage of their journey with the disease, and for 66 per cent of them the traditional healer was the first point of contact [21]. Medical pluralism, also known as the utilisation of multiple treatment modalities [22], occurs in circumstances where both biomedical and traditional healers are available to individuals [23]. Medical pluralism in Sudan reflects a postcolonial struggle between biomedicine’s hegemony and indigenous epistemologies [24]. For people with mycetoma, this manifests as fragmented care pathways shaped by mistrust in biomedical systems and cultural affinity for healers. The terms ‘sequential’ and ‘simultaneous’ describe two health-seeking approaches that are used by individuals to access healthcare services. Sequential health seeking occurs when a patient visits more than one healthcare provider, trying each new provider only after the previous one has failed to provide a diagnosis or effective treatment [25]. In contrast, simultaneous health seeking describes a patient’s seeking diagnosis and treatment from multiple healthcare providers at the same time [26].

Sequential health seeking is common among individuals with chronic illnesses, who may need long-term care [27]. However, sequential health seeking can lead to delayed diagnoses and treatment, as people may spend a significant amount of time seeking care from different providers before receiving effective treatment. In contrast, simultaneous health seeking is common among individuals seeking second opinions, or with those who lack confidence in the diagnosis provided by a single healthcare provider [27]. However, simultaneous health seeking can lead to fragmented care and increased healthcare costs, as patients may receive conflicting diagnoses and treatments from different providers.

Little light has been shed on the relationships between traditional healers and the biomedical treatment of mycetoma both globally and nationally in Sudan. All contemporary studies focused on traditional healing in the country are biomedical and quantitative in nature and were conducted within the Mycetoma Research Center (MRC) [21, 28, 29]. Within the field of neglected tropical diseases, the need for a biosocial approach is paramount, especially when the gap between knowledge and practice is significant and when behavioural changes are necessary [30].

The relationship between biomedical and traditional healing practices is complex and has long been a topic of interest within medical anthropology. Often, biomedical medicine has become dominant over traditional healing practices [31–33]. In this paper, we explore people’s health-seeking behaviours, the factors that influence these behaviours, and the reasons why people seek traditional healing treatment as a first treatment choice. We additionally examine, from a medical anthropological perspective, how the biomedical mycetoma treatment system in Sudan overlooks the traditional healing system. Providing evidence of the shortcomings of this neglect, we claim the need for, and suggest ways to, bridge the gap between the biomedical and traditional mycetoma healing systems in Sudan, in order to improve treatment outcomes and the overall wellbeing of those affected by mycetoma.

## Methods

Data was collected in the eastern part of Sennar State, Sudan. It was collected via rapid ethnographic fieldwork over periods of one week in 2018, one month in 2019, and three months in 2021. Our qualitative ethnographic approach involved participant observation. The initial observations focused on general, open-ended data collection, derived from individuals affected with mycetoma. In-depth interviews (both formal and informal), focus group discussions, participatory observations, and analyses of key documents were also conducted.

A total of 78 interviews were conducted: eight key informants were affiliated to the Federal Ministry of Health (FMOH), some of whom were from the Department of Communicable and Non-Communicable Disease Control, which is responsible for neglected tropical diseases (NTDs) in Sudan, while four government officials were affiliated to the Sennar State Ministry of Health where the village is affiliated. all of whom were related to the mycetoma programme in the state ministry. Additionally, a total of six interviews were conducted with staff members, physicians, and researchers from the MRC. (Table 1)

**Table 1:** Demographics table summarizing the method and participants

Method	Description	Participants/Subjects	Number of Interviews	Purpose/Focus
Key Informant Interviews	Semi-structured interviews with officials and experts in the field of mycetoma management and health policy.	8 key informants from the Federal Ministry of Health (FMOH) and 4 from the Sennar State Ministry of Health, including those in the mycetoma programme.	12	To gain insights from government health officials regarding mycetoma management and policies in Sudan.
Mycetoma Research Center Staff Interviews	In-depth Interviews with staff members, physicians, and researchers at the Medical Research Council (MRC).	6 individuals involved in mycetoma research and treatment at the Mycetoma Research Center.	6	To understand the medical research, perspectives, and treatment approaches related to mycetoma.
Community Member Interviews	In-depth interviews with local residents of Wad Elnimear village to gather knowledge, perceptions, and experiences.	30 members from the Wad Elnimear community.	30	To document the community’s knowledge, experiences, and perceptions regarding mycetoma.
Patient Interviews	Interviews with individuals diagnosed with mycetoma to explore their experiences and challenges.	20 patients with first-hand experience of mycetoma.	20	To gain an understanding of the patients’ journey, challenges, coping mechanisms, and treatment experiences.
Caregiver Interviews	Interviews with caregivers to understand their role in supporting individuals affected by mycetoma.	8 caregivers of individuals with mycetoma.	8	To capture the perspectives of caregivers and their roles in managing the disease at the household level.
Traditional Healers Interviews	Interviews with traditional healers to understand their practices and role in the local management of mycetoma.	2 traditional healers practicing in the region.	2	To explore the role of traditional healing practices in the management of mycetoma and their integration with biomedicine.
Environmental Observation	Field visit to East Sennar state to observe the socio-cultural environment, daily routines, and climate. as well as to review and analyse Mycetoma Research Centre (MRC) health promotion posters displayed in public and healthcare spaces.	N/A	N/A	To document the social, cultural, and environmental context of the study area—including the messaging and visual representation of mycetoma in health promotion materials—which influences disease management.

To ensure a comprehensive understanding of the experiences of the service users, thirty in-depth interviews were also conducted with individuals living in the communities of East Sennar State. These interviews allowed us to explore people’s knowledge, experiences, and perceptions regarding mycetoma. In addition to community members, interviews were conducted with community leaders who were closely associated with the area. Twenty interviews were conducted with patients who had first-hand experience of the disease, and which shed light on their journey, challenges, and coping mechanisms. Eight interviews were conducted with caregivers, who played a crucial role in supporting and caring for individuals affected by mycetoma. Lastly, insights from traditional healers were gathered through two interviews.

To document these findings, interviews were transcribed, key observations were recorded, and photographs and videos were taken to support the emerging analysis. The initial field engagement involved a short visit to investigate a project in East Sennar State, one of the areas in Sudan most highly endemic for mycetoma. This visit captured not only verbal accounts, but also the physical and social environment, including climate, social interactions, village events, and day-to-day routines.

The lead researcher is a medical doctor with prior collaborative work at the MRC and sustained engagement with affected communities in Sudan. This professional background facilitated rapport with participants and access to key informants, but also required reflexive attention to the potential influence of biomedical training and institutional affiliation on data interpretation. Recognising this positionality is important for situating the observations and analysis within both the researcher’s lived experience and the broader critical medical anthropology framework employed in this study.

The interview and observation data were analysed thematically using an inductive approach informed by Braun & Clarke’s six-phase method of thematic analysis. This involved familiarisation with the data, coding, developing, and refining themes, and interpreting them within a critical medical anthropology framework. Field notes and observations were triangulated with interview transcripts to enhance validity [34].

To systematically analyse the MRC’s educational posters (Figs. 3 and 4), we employed semiotic analysis [35] to understand how visual signs (e.g., colour, composition, symbols) construct meaning. The analysis focused on:

1. Representation: How traditional healers and doctors are depicted (e.g., clothing, skin tone, facial expressions).
2. Narrative: The implied storyline (e.g., suffering vs. cure).
3. Power Dynamics: Visual hierarchies (e.g., positioning, size).

### Ethical approvals

Ethical approvals were obtained from the Brighton and Sussex Medical School (BSMS), reference no. ER/BSMS9DHJ/9, and the Soba Center for Research and Audit, Soba Teaching Hospital, University of Khartoum IRB No. 27,122,018. We followed local Institutional review board protocol and informed the Research Governance and Ethics Committee at BSMS accordingly.

### Limitations

This qualitative study was conducted in a single rural, high-endemic area of Sudan, and this limits transferability of findings to other areas of the country. This approach however allowed us to acquire a deep understanding of the complex dynamics around mycetoma management in this area. We recognise that reliance on self-reported accounts may have introduced recall or social desirability bias; this was mitigated through cross-checking data across sources. In addition, the lead researcher’s background as a medical doctor with prior engagement at the MRC facilitated contextual insight but may have influenced data analysis and interpretation of findings. Reflexive journaling, peer debriefing, and verification of interpretations with local assistants were used to prevent this.

## Results

**Fig. 1:**
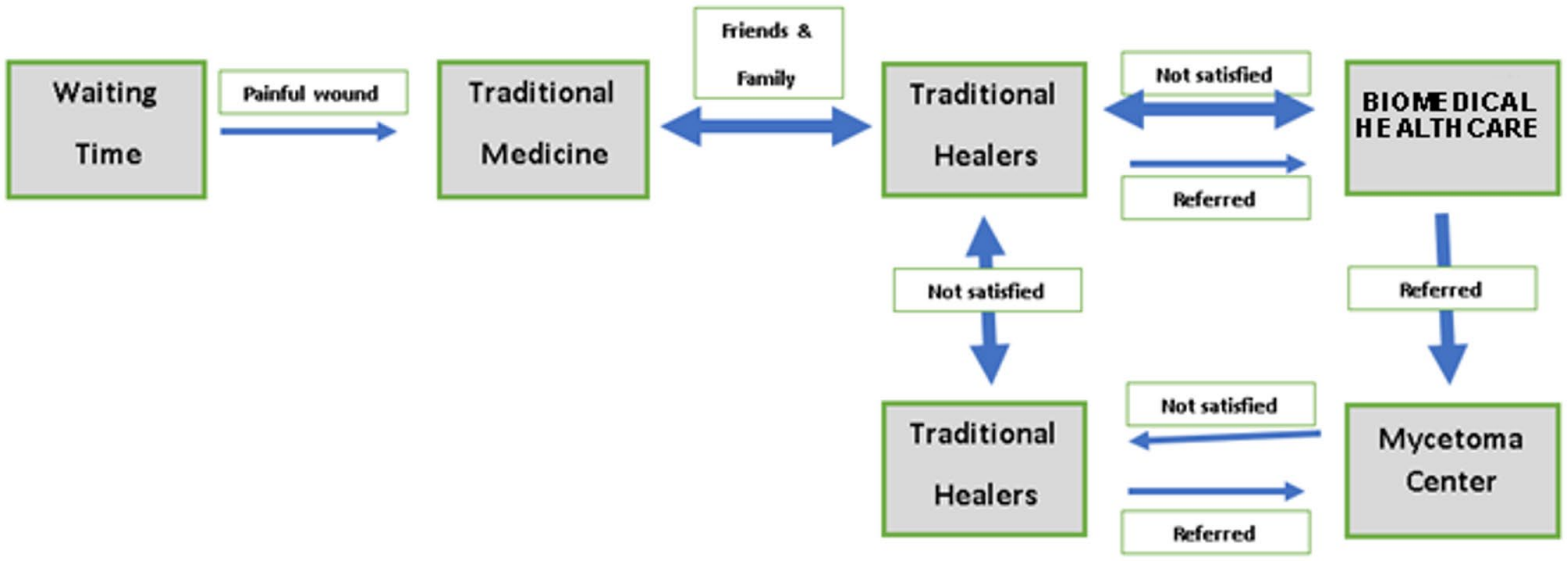
Mycetoma health-seeking pathways in Wad Elnimear village (prepared by the authors). The term “not satisfied” refers to cases where biomedical treatment was perceived as ineffective or insufficient in meeting patient expectations, which may reflect a mix of therapeutic outcomes, communication gaps, and culturally shaped perceptions of care. Notably, the direction of referral observed during the study was unidirectional—from traditional healers to biomedical providers—aligning with Kleinman’s [36] model of the professional, folk, and popular sectors, in which movement between sectors is shaped by both perceived efficacy and structural accessibility

Mycetoma starts with a painless lesion, so most individuals continue their life as usual without seeking immediate healthcare. Most of the individuals with the disease become worried only when the lesion turns painful and starts to limit their daily activities. Findings suggest that people use different mycetoma management pathways. Of those individuals who go directly to traditional healers, some remain with them exclusively, while others are referred by a healer to biomedical facilities. Data also indicates that various individuals with mycetoma sought treatment simultaneously from both biomedical and traditional healers, and some return to traditional healers after seeking treatment in biomedical centres. (See Fig. 1, below).

### Traditional healers are the first point of contact

The first healthcare encounter for those who have mycetoma is most frequently with a traditional healer; These healers use different modalities to treat mycetoma, including various herbal remedies, cauterisation of the wound, *ruqia* (recitation of specific verses from the Holy Quran), or *mehaya* (drinking water in which written Quranic verses have been dissolved), and other prayers (*duaa*) seeking relief from Allah.:

*’Firstly*,* [I was] injured due to acacia thorn and after [a] long time [the] wound exuded black matter*,* so I went to meet the sheik* [religious healer].’’

(30-year-old male, diagnosed with mycetoma)

**Fig. 2:**
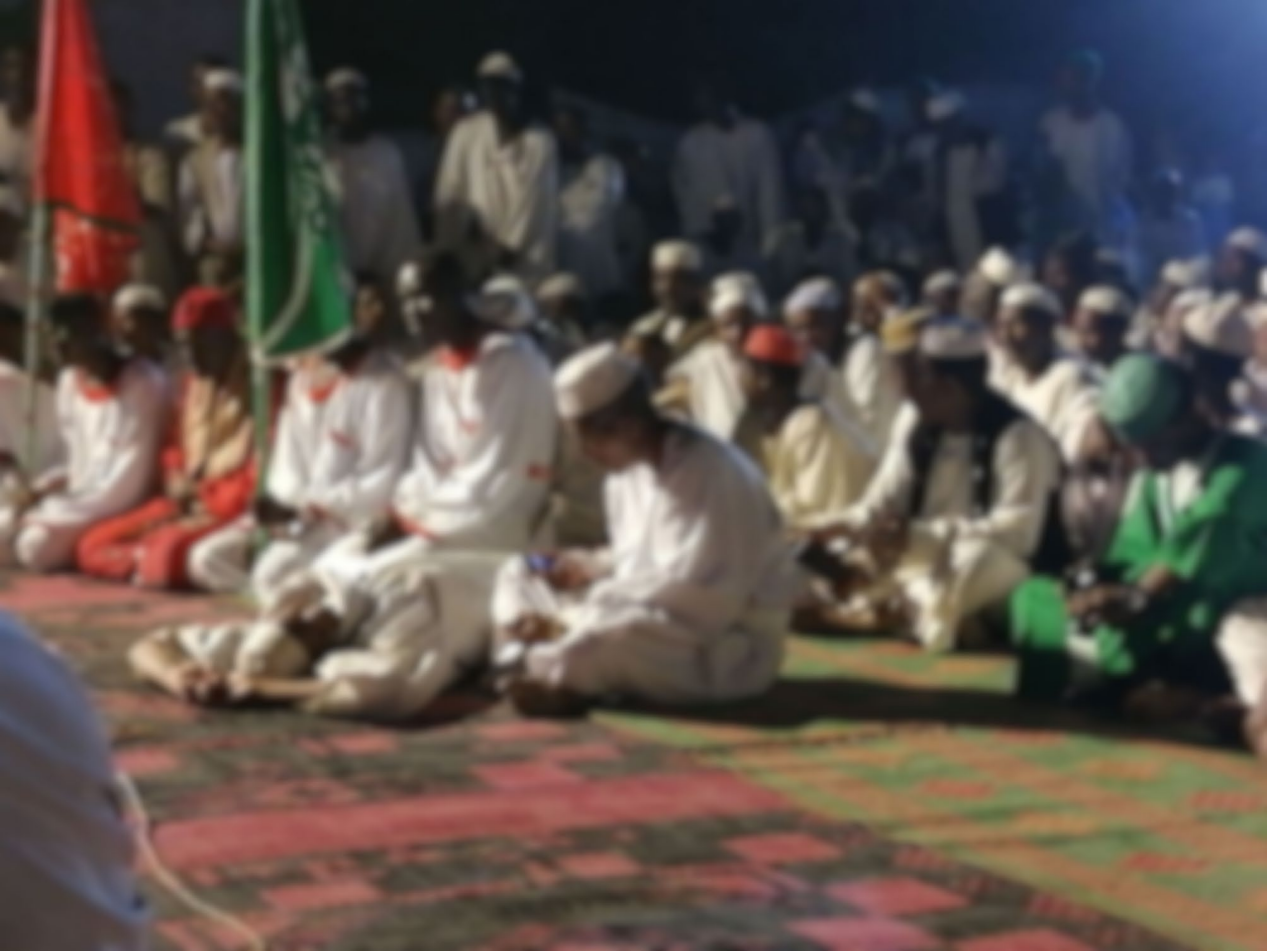
‘Alholia’, a large gathering for religious leaders in East Sennar State Photograph by the first author

Figure 2 below shows a *holia*, an annual gathering held to commemorate the anniversary of the *sheik* who founded a specific Sufi order. While the *holia* depicted here took place in East Sennar State, such events occur across Sudan in locations associated with particular Sufi leaders. These gatherings attract large numbers of attendees, many travelling long distances, including those seeking *duaa* in the hope of finding relief from medical conditions. Government officials were also present at this event in a ceremonial capacity, offering formal recognition of the gathering’s cultural and social significance.

Most of these individuals reported accessing every possible treatment modality, including every available traditional healer and form of biomedical medicine, because, they admitted, they were desperate and dissatisfied with the treatment outcome of each previously tried treatment system:

’He visited every single sheik, Abdella, Wad Alobaid, all of them. He tried every single powder and every single doctor.’’

(45-year-old female, wife of individual with mycetoma)

’If someone told him about [a] sheik, that he could possibly treat him, he would immediately go and see that sheik.

(38-year-old female, wife of individual with mycetoma)

*‘’Alsheik Alsammani told him to grind kamoon – ‘black seed’ – mix it and apply it every morning and every night; he tried it and said it didn’t work; it only made his clothes dirty. He wanted to try it once or twice to see if it worked*,* then he went to Soba teaching hospital*,* and they told him*,* “Your hand is sprouting”.*

(35-year-old female, wife of individual with mycetoma)

I went three times and it refused to heal; I went to a hospital in Madani to see a doctor, the doctor operated on me and told me to come back after a while. It came back again aggressively with pain. After Madani my body became weak, my hair broke off, and I became very skinny – people did not recognise me because my hair was white, I vomited all the time. I was very distressed, then my brother decided that we should go to Khartoum.

(37-year-old male, diagnosed with mycetoma)

### One health system at a time

Some participants indicated that while being treated by a traditional healer, they would never try using biomedical pills or prescriptions, nor even visit a doctor. This was because they believed that they should try only one system of health-management at a time, and this often also reflected the instructions of the traditional healer. A similar finding was reported by Kunna et al. (2021c).

*‘’I tell him/her to stop taking [the] doctor’s medication for one week – I need to see if there is improvement or not*,* then I can advise him/her accordingly. In some diseases the medication works*,* in others it is not working’’.*

(49-year-old male, traditional healer)

### Referrals from traditional healers

It was not uncommon for individuals with mycetoma to be referred by their traditional healers to seek biomedical treatment. During one of my fields visits I witnessed how a traditional healer was following their progress by phone and how both individuals with mycetoma expressed an interest in travelling to the (MRC) in Khartoum for biomedical treatment. Traditional healers do care about their own reputation and the curing of their patients, as one of the traditional healers stated:

’There are people who have infection, I do not cauterise them! Instead, I tell them to go to the doctors and do some investigation. Cauterisation is not for all cases. For some conditions cauterisation will not work – they [had] better go to the doctors.

(49-year-old male, traditional healer)

’Those who have mycetoma I do not cauterise them; I can differentiate it from other similar conditions like joint dislocation – they come with football injury or fall down on their foot.

(49-year-old male, traditional healer)

### The preference for traditional healers over biomedical healthcare in Mycetoma treatment

Multiple complex factors play interconnected roles in shaping decisions about when, where, and from whom to seek treatment for mycetoma. These include economic and geographic barriers (transport costs, distance to Khartoum, lack of PHC services), cultural and spiritual beliefs (perceptions of causation and reliance on ruqia, mehaya, and prayers), perceptions of efficacy and trust (dissatisfaction with biomedical treatment, reputation of healers and doctors, and advice to use one system at a time), and broader structural and social constraints (weak health-system protocols, donor neglect, stigma, and gendered vulnerabilities. The following subsections discuss the factors most prominent in shaping participants’ accounts of health-seeking and treatment decisions.

#### Economic and Geographic Barriers

For many patients the lengthy return trip to the MRC is prohibitively expensive, and travel costs are exacerbated by the instability of the currency and the general economic situation in Sudan. Further, the stay in Khartoum State required to fulfil each follow-up visit to the MRC incurs additional costs. Mycetoma patients must visit the centre for further treatment and consultations every six weeks, prior to which the patients are also required to do a liver function test. One of the patients in the study was obliged to stay with her married sister for 10 days after each follow-up visit, during which she was unable to attend school, adversely affecting her education.

During interviews, one of the patients reported that he visited the centre mainly to obtain the free medication they provided (which is supported by the Federal Ministry of Health (FMOH)):

’I just visit the Mycetoma Research Center for the medication – it’s very expensive outside the MRC.

(30-year-old male, diagnosed with mycetoma)

Although their economic situation and the travel distance to the MRC were the reasons most frequently cited by the participants, these were not necessarily the most important factors in the decision to seek a traditional healer.

Some patients who lived close to a traditional healer were nevertheless willing to travel long distances to reach a different traditional healer.

*‘’I heard about one of the traditional healers from my friend*,* in Nuba Mountain in western Sudan. I visited him seeking treatment for my foot’’.*

(34-year-old male, diagnosed with mycetoma)

One of the Mycetoma patients consulted during this study travelled approximately 300 km from Sennar State to Khartoum to seek care from a traditional healer. This decision appeared to be driven less by cost than by the patient’s perception that the healer was more likely to provide effective treatment. Such perceptions of efficacy, shaped by personal experience and community narratives, influenced many patients’ therapeutic choices.

#### Dissatisfaction with Biomedical Care

While a small number of patients and families reported satisfaction with biomedical care—particularly when early diagnosis and timely surgical or pharmaceutical intervention were achieved—several others described frustrations with biomedical treatment. These frustrations, which often stemmed from perceived ineffectiveness, side effects, and strained communication with providers, prompted some patients to seek or return to traditional healers.

Many patients felt dissatisfaction with the MRC, and this presented in many ways:

*‘’They do not do anything [referring to the MRC]*,* they do not treat the disease*,* and they do not amputate it’’.*

(37-year-old male, leg amputated in a hospital due to mycetoma)

’The doctor gave me the white pills. For three years I kept taking them and attending the follow-up every six weeks in vain. I got so tired with my leg. I gave them back their pills and informed them I am not going back to that doctor [after] three years of ineffective treatment. Nothing but the pills.

(28-year-old male, diagnosed with mycetoma)

This account reflects a common frustration among patients—limited awareness that biomedical treatment for mycetoma can require prolonged, sustained adherence before visible improvement occurs. Such perceptions often lead to treatment discontinuation and reversion to traditional healing. This underscores the importance of early detection and timely intervention, which can shorten treatment duration and improve outcomes. Traditional healers, as the first point of contact for many patients, could play a pivotal role in recognising symptoms early and facilitating prompt biomedical referral.

*‘’We approached the director of the MRC in his private clinic seeking his referral letter to travel to Egypt*,* seeking management there’’.*

(53-year-old male, father of individual with mycetoma)

#### Social Support as Care Determinant

Speaking about their experiences in one of the central hospitals, most patients positively valued the social support they received there; one patient proudly reported:

When I went in to [the] operation, there were 20 of my relatives outside and 20 inside the hospital.

(30-year-old female, diagnosed with mycetoma)

One patient reported that this level of social support could not be sustained when they travelled to Khartoum for treatment through the MRC:

’I do not have relatives in Khartoum, I only have my brother [who] works there; I’m very grateful to his neighbour [that] they allow me to spend one week, but still, it’s not like when you live in your relatives’ house.

(38-year-old female, diagnosed with mycetoma)

Some of the patients approached traditional healers even after being treated through the MRC because they were dissatisfied with the treatment outcomes:

*After his first surgical operation*,* he went to the MRC for follow-up several times; every time they asked him to go back and come again …. He was never convinced*,* then he returned to using the remedies – he carried his remedies in his bag when going to Khartoum to [the] MRC [he was using both traditional and biomedical treatment].*

(38-year-old female, wife of individual with mycetoma)

### The perspective of healthcare providers in MRC

In all of the studies carried out by MRC staff, traditional healers have been viewed as a major contributor to delays in patients seeking biomedical treatment through the MRC [21, 28, 29]. From among these studies, only one acknowledged the importance of cooperation with traditional healers, and even then such collaboration was framed primarily as a means to accelerate referrals to the MRC [21]. This framing is likely to entrench a predominantly negative attitude towards traditional healers and their practices, which may make genuine collaboration challenging.

When ‘cooperation’ is advanced primarily as a strategy to boost early identification and increase referrals, it risks overlooking the broader cultural and therapeutic roles of traditional healing within communities. Similar patterns have been observed in other African contexts, such as in mental health collaborations in Kenya [37] and community mental health task-shifting initiatives in Kerala, India [38] where tensions and ruptures arose when collaboration was framed through a one-directional biomedical lens. These dynamics suggest that without mutual recognition of legitimacy, power imbalances will continue to limit the scope and sustainability of biomedical–traditional healer partnerships.

Not only do doctors at the MRC attribute delays in patients’ access to biomedical care and treatment pathways to the role of traditional healers, but they also view the MRC as the most appropriate place to treat mycetoma patients because, from a biomedical perspective, it is perceived as offering the most effective treatment. The provision of medication free of charge, supported by the Federal Ministry of Health (FMOH), is seen as an additional measure to promote access, rather than the primary reason for considering the MRC the optimal treatment site. Some physicians further attribute patients’ reluctance to seek treatment at the MRC to economic constraints, including the cost of travel and accommodation during prolonged treatment courses.

*‘’Most of the mycetoma patients arrive at the centre very late; they take much time with traditional healers and taking home remedies. This delay may raise the possibility of having surgical intervention and maybe even amputation’’.*

(MRC doctor)

*‘’If they have money*,* they will immediately come to the centre*,* they will never approach the traditional healers’’.*

(MRC doctor)

### System-level barriers and policy perspectives (FMOH)

Interviews with officials at the Federal Ministry of Health (FMOH) revealed how systemic and structural gaps reinforce patients’ reliance on traditional healing. Several informants acknowledged that, despite mycetoma’s recognition as a neglected tropical disease, it lacks clear national protocols at the primary healthcare (PHC) and rural hospital levels. This gap leaves frontline providers uncertain about how to identify and manage suspected cases, resulting in delays and over-reliance on tertiary referral. As one FMOH key informant explained:

We drafted a proposal for the survey… it was made with the mindset of a surgeon; aiming not to put a single hand on the patient, but just for high-level detection of the disease and referral. There is no protocol for rural hospitals nor at the PHC level.

### (Key informant, FMOH)

This system-level neglect was compounded by donor-driven priorities: while malaria, tuberculosis, and HIV programs received sustained funding, mycetoma was consistently excluded. As another FMOH informant explained, *“The fund is only allocated to Malaria*,* TB and HIV… NTDs like mycetoma are not killers*,* they are disabling — so they receive no budget.”* Such policy-level dynamics illuminate why patients often seek traditional healers first, or cycle between therapeutic systems, given the limited structural support for mycetoma within Sudan’s formal health system. Such testimonies resonate with wider evidence that top-down biomedical interventions often fail when they do not account for local realities [39]. These patterns echo findings from an earlier study where villagers resisted a government-endorsed prevention project, revealing tensions between state-led biomedical logics and community lifeworld [40].

### Representations & symbolism

**Fig. 3:**
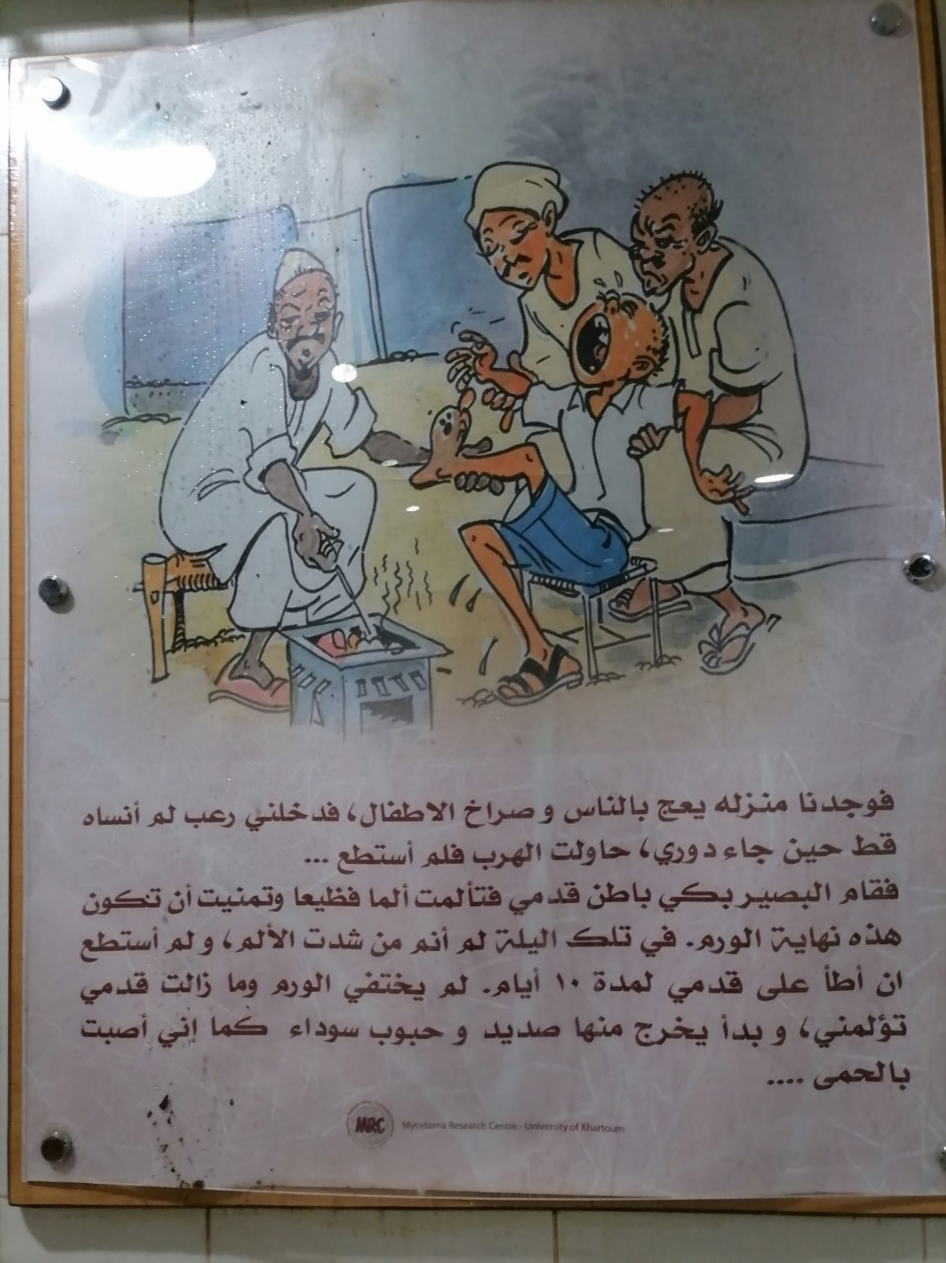
MRC poster depicting a boy being taken by his family to a traditional healer

**Fig. 4:**
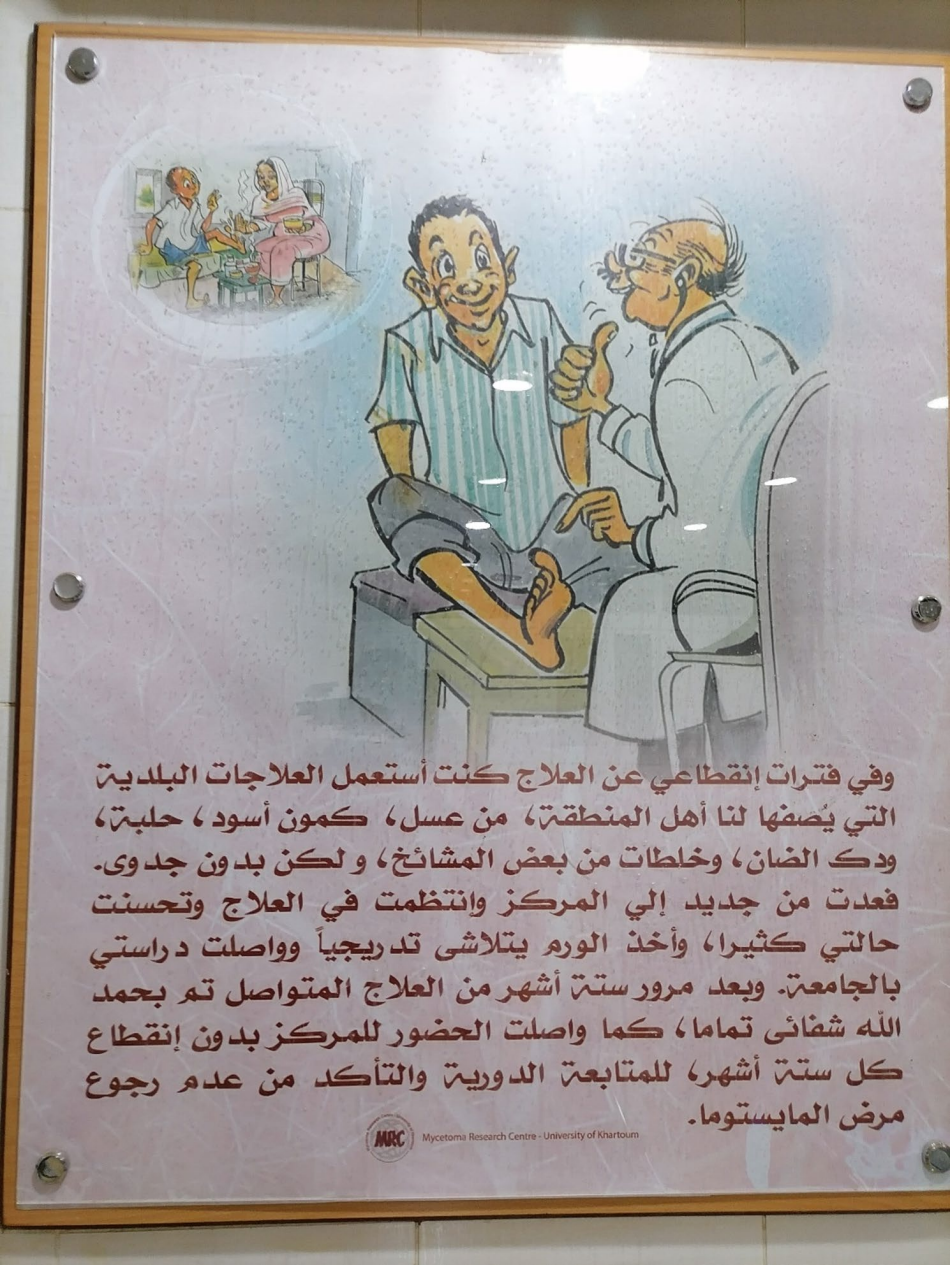
MRC poster depicting a young man’s visit to an MRC doctor

Semiotic analysis revealed that the MRC posters (Figs. 3 and 4) perpetuate colonial hierarchies through racialized imagery and narrative framing. The healer’s darker skin and traditional attire contrast with the doctor’s western appearance, visually equating biomedicine with progress and healers with regress [41]. This rhetoric risks alienating patients who value cultural congruence in care.

Figure 3 (above) shows a poster that is displayed in the MRC waiting area. The vignette tells the story of a little boy taken to visit a traditional healer; the text, written in Arabic, says:

*We [the boy and his family] found his [the traditional healer’s] house full of people and the sounds of crying boys. I panicked when it was my turn and will never forget that fear ever in my life – I tried to escape but was unable to do so.*

*Then the healer cauterised the sole of my foot; I felt terrible pain and wished for the end of this swelling. I didn’t sleep that night*,* and I couldn’t walk for 10 days. The swelling didn’t go away*,* and my foot was still painful. In addition*,* there were pus and black grains coming out from the wound and I had a fever.*

The poster in Fig. 4, also displayed in the MRC waiting room, shows the scene of a young man who, the writing states, reports having previously tried alternative herbal remedies prescribed by traditional healers and sheiks; none of these alternatives helped his mycetoma, he says, but when he returned to the MRC after visiting the traditional healer, his disease was completely cured after six months of continuous management there. The writing continues by saying that the young man was also able to commence study at university once the swelling had gone, and he was only attending for a follow-up visit every six months to ensure the disease did not return.

The contrasting illustrations can be seen as reflecting to some extent the views of the MRC towards traditional healers: the healer is depicted as a sad, ugly, and unsmiling person, and is given an Arabian look with darker skin-tone, an Arab-style moustache, and a ‘meaner’ overall look, in contrast with the lighter skin-tone and more ‘western’ appearance of the MRC doctor, who is shown as friendly and welcoming. This may indicate an underlying assumption that a ‘Western’-looking doctor is more trustworthy than an Arabian-looking traditional healer. Furthermore, the traditional healer is associated with the negative emotions of suffering, pain, and fear, while the MRC doctor is shown in a context of positive emotion, with regret only for ever having visited a traditional healer. In addition, the posters communicate the idea that disease recurrence is associated only with traditional healers and their remedies, while at the MRC, in contrast, a complete cure is possible after only six months of continuous treatment and follow-up.

Another observation that has been made in the context of the MRC is the potential impact on the patient experience of the traditional ‘formal white coat’ worn by doctors. While some studies recommend the wearing of a white coat to create an image of authority, attractiveness, and trust [42], in the context of the MRC, the presence of the white coat may create a perception of authority and power and facilitate a power dynamic between the physician and patient. This dynamic can be understood as part of the larger history of marginalisation and devaluation of traditional healing practices by Western biomedical models; as a symbol of Western biomedical authority, the white coat can serve to reinforce this dynamic and widen the divide between Western biomedical practitioners and traditional healers.

To address this issue, it may be important to consider alternative approaches to the physician–patient relationship that prioritise collaboration and mutual respect. This may involve rethinking the attire worn by doctors, as well as adopting more inclusive and culturally sensitive practices that acknowledge and value the role of traditional healing practices in the context of mycetoma treatment. By working to bridge the gap between the practices of Western biomedical and traditional healing systems, it may be possible to improve the patient experience and outcomes for those affected by mycetoma.

The MRC posters discussed above illustrate how the MRC not only stigmatises traditional healers and views them as a fundamental cause for delay in the management of mycetoma disease but may even act against them. (Table 2 )presents all themes and codes identified across patients, families, traditional healers, MRC providers, and FMOH officials, together with illustrative quotations.

**Table 2:** List of themes, codes, and illustrative quotations on Mycetoma Health-Seeking and management pathways in East Sennar state

Theme	Codes Identified	Illustrative Quotes
Health-seeking decisions	Delay until pain; initial reliance on traditional healers; exclusive reliance on one system; moving between systems; referrals from healers to MRC	“Firstly, [I was] injured due to acacia thorn… so I went to meet the sheik.” (30-year-old male, diagnosed with mycetoma) “I tell him/her to stop taking the doctor’s medication for one week – I need to see if there is improvement or not.” (49-year-old male, traditional healer)
Traditional healing practices	Herbal remedies; cauterisation; ruqia & mehaya; duaa; trial of remedies from multiple healers	“Alsheik X told him to grind kamoon – black seed – mix it and apply it every morning and night; it didn’t work.” (35-year-old female, wife of patient)
Referrals and healer strategies	Referrals to biomedical care; healer reputation management; diagnostic discernment by healers	“There are people who have infection, I do not cauterise them! Instead, I tell them to go to the doctors.” (49-year-old male, traditional healer)
Economic & geographic barriers	Travel costs; accommodation in Khartoum; lost school/work time; medication affordability	“I just visit the Mycetoma Research Center for the medication – it’s very expensive outside the MRC.” (30-year-old male, diagnosed with mycetoma)
Perceived (in)efficacy of biomedical care	Dissatisfaction with prolonged treatment; perception that doctors “do nothing”; side effects; recurrence	“The doctor gave me the white pills… after three years I gave them back and told them I am not going back.” (28-year-old male, diagnosed with mycetoma)
Social support as care determinant	Family accompaniment during surgery; lack of kin support in Khartoum	“When I went into operation, there were 20 of my relatives outside and 20 inside.” (30-year-old female, diagnosed with mycetoma)
Simultaneous use of systems	Carrying remedies while attending MRC; combining healer remedies with biomedical pills	“He was never convinced, then he returned to using the remedies – he carried his remedies in his bag when going to Khartoum.” (38-year-old female, wife of patient)
Provider perspectives (MRC)	Healer’s cause delay: belief that money would draw patients sooner; advocacy of MRC as “best” site	“Most of the mycetoma patients arrive at the centre very late; they take much time with traditional healers.” (MRC doctor)
Symbolic/representational issues	Posters portraying healers negatively; biomedical “white coat” as authority symbol	“The healer is depicted as a sad, unsmiling person with darker skin, in contrast with the friendly, western-looking doctor.” (Observation of MRC poster)
Policy & system-level barriers (FMOH)	Lack of PHC protocol; survey proposal shaped by surgical mindset; donor neglect; absence of prevalence data	“We drafted a proposal for the survey… it was made with the mindset of a surgeon… there is no protocol for rural hospitals nor at the PHC level.” (Key Informant, FMOH) *“The fund is only allocated to Malaria, TB and HIV… NTDs like mycetoma are not killers, they are disabling — so they receive no budget.”*

## Discussion

Traditional healers play a major role in the journey of mycetoma patients, with most individuals seeking care from them either before or alongside biomedical care. As shown, in addition to financial reasons, individuals’ healthcare decisions were associated with various other factors, including dissatisfaction with the outcome of specific treatment modalities and the need for social support, lack of which may present as a major obstacle in patients’ obtaining biomedical treatment. This is supported by hypotheses which state that social relationships influence individuals’ health and wellbeing under all conditions (Loucks et al., [43]), and buffering hypotheses, which suggest that social relations are predominantly influential during times of stress [44].

Largely because there is still neither a definitive treatment nor a conclusive diagnostic modality for mycetoma [45, 46], individuals with mycetoma tend to seek whatever treatment they are able to access. We used the concepts of *sequential* and *simultaneous* healthcare-seeking pathways to describe the different strategies used by people with mycetoma. The chronicity of the disease, the lengthy duration of the biomedical treatment, the medication’s side effects, and the high rate of recurrence all were found to influence individuals’ health-seeking behaviours.

As shown, the power dynamics between biomedicine and traditional healing practices are complex and multifaceted. These hierarchies of knowledge play a vital role in individuals’ decisions, and this is especially true for marginalised and rural communities, who may feel excluded or mistreated by biomedical healthcare systems, especially if they had tried to manage the disease via traditional methods. In turn, individuals who feel disempowered may be more likely to seek alternative healing practices.

Traditional healing practices have been marginalised and devalued by biomedical medicine, in this case in the form of the MRC. This marginalisation has historically been driven by several factors, including the colonisation of many parts of these institutions by Western powers, the influence of Western pharmaceutical companies, and the dominance of biomedical Western medical practices within health institutions [47, 48].

The marginalisation of traditional medical practices has led to the neglect of local knowledge and healing systems that, for centuries, have long been central to the health and wellbeing of many communities in Sudan. These practices are culturally specific and inclusive of contextual beliefs and values. For example, individuals who visit the MRC seeking biomedical healthcare often lament the lack of social support, which drives them to seek treatment from traditional healers. In this context, understanding the complex interplay between cultural beliefs, social support, and healthcare-seeking behaviour is critical. This finding aligns with those of other qualitative studies focused on the management of neglected tropical diseases of the skin in Central America. For instance, intercultural communication barriers also significantly impact the effectiveness of cutaneous leishmaniasis control efforts in Guatemala [49, 50]. In these studies, authors highlighted that local misunderstanding of the disease, combined with cultural stigma, often deterred individuals from seeking timely treatment, while health workers’ lack of cultural competence hampered the delivery of quality healthcare services [49]. These challenges are exacerbated by resource constraints, leading to gaps in surveillance and treatment [50]. This example from Guatemala emphasizes how critical it is to consider cultural and communication factors when designing public health interventions, a lesson that could also be applied in efforts to combat neglected diseases like mycetoma in Sudan.

As shown, the dominance of the MRC’s biomedical approach has negatively impacted the doctor–patient relationship in the context of mycetoma management in Sudan. This dominance is symbolised by the traditional *white coat* worn by MRC doctors, a garment widely associated with Western medical professionals. Field observations in Sudanese public hospitals made clear that white coats were a visual marker of biomedical authority. This attire, in turn, reinforces a hierarchical power dynamic between doctors and patients and, although more research is needed in this area, our research suggests that may contribute to a breakdown in communication and trust. These dynamics are further exacerbated by the MRC’s position as a westernised institution that may not fully understand or respect local cultural beliefs and practices. Such dominance is also evident in the fact that free medication for mycetoma is available only under the MRC’s authority and in its formal connections with Western institutions such as the WHO.

Importantly, compared with biomedical healthcare providers, traditional healers interact rather differently with their patients. Traditional healers often use aa more people-centred approach, focusing on a more holistic manner on psychosocial and daily life issues together with health issues. They also solicit their patients’ opinions more frequently and engage in discussions about patients’ own concepts of illness [12]. Indeed, these approaches build trust.

In what can be seen as a form of cultural imperialism, wherein biomedical practices are promoted as superior to traditional medical practices, the MRC not only stigmatises traditional healers but also actively works against their medical system. The negative portrayal of traditional healers is evident in the posters found displayed inside the centre, in which the healer is depicted as sad, ugly, and non-smiling; in contrast, the MRC doctor is shown as welcoming and friendly, and as having a relatively lighter skin-tone and a more modern-looking appearance, which may also reflect an underlying assumption that foreign doctors are more trustworthy than traditional healers. The posters also imply that seeking treatment from traditional healers can result in further suffering and delay in the ‘proper’ management of mycetoma. As shown, these representations frame the traditional healer as a problem or barrier to effective treatment, rather than recognising their potential as a partner or facilitator within a collaborative care approach.

Some researcher have argued that in Africa it is mainly poor, marginalised, and less-educated people who seek care from traditional healers, and that they do so because these healers offer treatment at lower cost and are easier to access [12]. As shown, this assumption is also held by many of the providers of biomedical Mycetoma treatment. Our study challenges these assumptions and shows instead that many other factors contribute to these treatment choices, including social support needs, cultural relevance and accessibility. As shown, some individuals affected by mycetoma travelled long distances to seek treatment from traditional healers. Epilepsy studies in Cameroon have also shown that individuals deliberately choose traditional care, even though it is 20 times more expensive than biomedical treatment [51, 53,52 ].

These findings may encourage policymakers, programme managers, and healthcare providers within the public health system to integrate patient-centred communication as a core component of care, while also fostering improved communication between the biomedical and traditional health sectors. Achieving this would require greater openness and transparency about each sector’s approaches and practices, with the aim of building mutual trust and a respectful working relationship. Such developments could improve the quality of care, enhance patients’ perceptions of healthcare, and strengthen the effectiveness of public health programmes for the prevention and management of mycetoma and other neglected tropical diseases in Sudan and across Africa.

## Conclusion

The role of traditional healers in the management of mycetoma disease in Sudan is important. Historically managed via traditional healing practices, mycetoma is now managed by both traditional and biomedical methods, but the authoritative dominance of biomedical medicine has often led to the marginalisation and stigmatisation of traditional healing practices, and often also of those who use these. This has, in turn, negatively impacted doctor–patient relationships, as people may feel uncomfortable with biomedical practices and prefer treatments that better align with their cultural beliefs and practices. While biomedical treatments may be effective in some cases, it is crucial to recognise the importance of traditional healing practices and the need to integrate them into the formal healthcare system. This can be achieved through building trust and collaboration between traditional healers and biomedical professionals, as well as by creating a more inclusive and culturally sensitive healthcare system. Our research indicates that this can lead to improved health outcomes and overall doctor-patient relations.

## Data Availability

Restrictions apply to the availability of these data, which were used under license for the current study, and so are not publicly available. Data are however available when emailing NTD.DataManager@bsms.ac.uk, subject to approval by the study PI.

## References

[1] Kirmayer LJ. The cultural diversity of healing: meaning, metaphor and mechanism. Br Med Bull. 2004;69:33–48. 15226195 10.1093/bmb/ldh006

[2] Mutale W, Matenga TFL, Wagner R, Clemens E, Audet CM. Integrating traditional healers into the health care system: challenges and opportunities in South Africa. 2020;47(4):305–12.

[3] El Safi, Ahmed. Traditional Sudanese Medicine : A Primer for Health Care Providers, Researchers, and Students. Khartoum, Sudan: AZZA House, 2007. Print. https://catalog.nlm.nih.gov/permalink/01NLM_INST/m5fc0v/alma9913068163406676.

[4] De Graaff A, Cuijpers P, Leeflang M, Sferra I, Uppendahl J, Sijbrandij M, et al. A systematic review and meta-analysis of the diagnostic accuracy of self-report screening instruments for common mental disorders in Arabic-speaking adults. Eur Psychiatry. 2022;65(S1):S94–5. 10.1017/gmh.2021.39 PMC8679833 34966543

[5] Moodley J, Pattinson RC, Baxter C, Sibeko S, Abdool Karim Q. Strengthening HIV services for pregnant women: an opportunity to reduce maternal mortality rates in Southern Africa/sub-Saharan Africa. BJOG: An International Journal of Obstetrics & Gynaecology. 2011;118(2):219–25. 10.1111/j.1471-0528.2010.02726.x. 21159120 10.1111/j.1471-0528.2010.02726.x

[6] Kennedy A, Sehgal A, Szabo J, McGowan K, Lindstrom G, Roach P, et al. Indigenous strengths-based approaches to healthcare and health professions education – Recognising the value of elders’ teachings. Health Educ J. 2022;81(4):423–38. 35531386 10.1177/00178969221088921 PMC9066669

[7] Kahissay MH, Fenta TG, Boon H. Beliefs and perception of ill-health causation: A socio-cultural qualitative study in rural North-Eastern Ethiopia. BMC Public Health [Internet]. 2017;17(1):1–10. Available from: 10.1186/s12889-017-4052-y 10.1186/s12889-017-4052-y PMC5267452 28122606

[8] Stekelenburg J, Jager BE, Kolk PR, Westen EHMN, Kwaak A, Van Der, Wolffers IN. Health care seeking behaviour and utilisation of traditional healers in kalabo, Zambia. Health Policy (New York). 2005;71(1):67–81. 10.1016/j.healthpol.2004.05.008 15563994

[9] Tabi MM, Powell M, Hodnicki D. Use of traditional healers and modern medicine in Ghana. Int Nurs Rev. 2006;53(1):52–8. 16430761 10.1111/j.1466-7657.2006.00444.x

[10] Ikhoyameh M, Okete WE, Ogboye RM, Gbadebo OS, Owoyemi OK. Integrating traditional medicine into the African healthcare system post-Traditional medicine global summit: challenges and recommendations. Pan Afr Med J. 2024. 10.11604/pamj.2024.47.146.43011. 38933435 10.11604/pamj.2024.47.146.43011 PMC11204987

[11] Kwame A. Integrating traditional medicine and healing into the Ghanaian mainstream health system: voices from within. Qual Health Res. 2021. 10.1177/10497323211008849. 33980093 10.1177/10497323211008849 PMC8446885

[12] Labhardt ND, Aboa SM, Manga E, Bensing JM, Langewitz W. Bridging the gap: how traditional healers interact with their patients. A comparative study in Cameroon. Trop Med Int Health. 2010;15(9):1099–108. 20545920 10.1111/j.1365-3156.2010.02575.x

[13] Oyebode O, Kandala NB, Chilton PJ, Lilford RJ. Use of traditional medicine in middle-income countries: a WHO-SAGE study. Health Policy Plan. 2016;31(8):984–91. 27033366 10.1093/heapol/czw022 PMC5013777

[14] Abdalla S, Aziz MA, Basheir I. Seeking care from a traditional healer after injury in Sudan: an exploratory cross-sectional analysis. Int Health. 2020;12(3):177–83. 32374407 10.1093/inthealth/ihz063 PMC11973418

[15] Fahal AH. Mycetoma. A thorn in the flesh. Trans R Soc Trop Med Hyg. 2004;98(1):3–11. 14702833 10.1016/s0035-9203(03)00009-9

[16] van de Sande W, Fahal A, Ahmed SA, Serrano JA, Bonifaz A, Zijlstra E. Closing the mycetoma knowledge gap. Med Mycol [Internet]. 2017 Sep 9 [cited 2017 Nov 1]; Available from: http://academic.oup.com/mmy/article/doi/10.1093/mmy/myx061/4108713/Closing-the-mycetoma-knowledge-gap 10.1093/mmy/myx061 28992217

[17] van de Sande WWJ. Global burden of human mycetoma: a systematic review and meta-analysis. PLoS Negl Trop Dis. 2013;7(11):e2550. 10.1371/journal.pntd.0002550. 24244780 10.1371/journal.pntd.0002550 PMC3820768

[18] Emery D, Denning DW. The global distribution of actinomycetoma and eumycetoma. PLoS Negl Trop Dis [Internet]. 2020;14(9):1–13. Available from: 10.1371/journal.pntd.0008397 10.1371/journal.pntd.0008397 PMC7514014 32970667

[19] Hassan R, Deribe K, Fahal AH, Newport M, Bakhiet S. Clinical epidemiological characteristics of mycetoma in Eastern Sennar locality, Sennar State, Sudan. PLoS Negl Trop Dis. 2021. 10.1371/journal.pntd.0009847. 34898611 10.1371/journal.pntd.0009847 PMC8699598

[20] Fahal A, Mahgoub ES, Hassan EL, Abdel-Rahman AM, Alshambaty ME, Hashim Y et al. A,. A New Model for Management of Mycetoma in the Sudan. PLoS Negl Trop Dis [Internet]. 2014 [cited 2021 Jan 20];8(10). Available from: /pmc/articles/PMC4214669/?report = abstract 10.1371/journal.pntd.0003271 PMC4214669 25356640

[21] Kunna E, Yamamoto T, Fahal A. The use of traditional medicines among mycetoma patients. Trans R Soc Trop Med Hyg [Internet]. 2021;115:297–306. Available from: https://academic.oup.com/trstmh/article/115/4/297/6008085 10.1093/trstmh/traa135 PMC8046407 33247308

[22] Leslie C. Medical pluralism in world perspective. Soc Sci Med Med Anthropol. 1980;14 B(4):191–5. 10.1016/0160-7987(80)90044-7 7209590

[23] Sundararajan R, Mwanga-Amumpaire J, King R, Ware NC. Conceptual model for pluralistic healthcare behaviour: results from a qualitative study in Southwestern Uganda. BMJ Open. 2020;10(4):1–11. 10.1136/bmjopen-2019-033410 PMC7204928 32317259

[24] Langwick S. The Value of Secrets: In: Olsen WC, Sargent C, editors. African Medical Pluralism [Internet]. Indiana University Press; 2017. pp. 31–49. Available from: http://www.jstor.org/stable/j.ctt1zxz1b8.4

[25] Haenssgen MJ, Ariana P. Healthcare access: A sequence-sensitive approach. SSM - Popul Heal. 2017;3(November 2016):37–47. 10.1016/j.ssmph.2016.11.008 PMC5769022 29349202

[26] Chomi EN, Mujinja PG, Enemark U, Hansen K, Kiwara AD. Health care seeking behaviour and utilisation in a multiple health insurance system: does insurance affiliation matter? Int J Equity Health. 2014;13(1):1–11. 24645876 10.1186/1475-9276-13-25 PMC3994926

[27] Wu S, Du S, Feng R, Liu W, Ye W. Behavioral deviations: healthcare-seeking behavior of chronic disease patients with intention to visit primary health care institutions. BMC Health Serv Res [Internet]. 2023;23(1):1–14. Available from: 10.1186/s12913-023-09528-y 10.1186/s12913-023-09528-y PMC10185376 37189156

[28] Elkheir LYM, Haroun R, Awadalla M, Id M, Id HF. Madurella mycetomatis causing eumycetoma medical treatment: The challenges and prospects. 2020;1–17. Available from: 10.1371/journal.pntd.0008307 10.1371/journal.pntd.0008307 PMC7452721 32853199

[29] Ezaldeen EA, Fahal AH, Osman A. Mycetoma herbal treatment: the Mycetoma research centre, Sudan experience. PLoS Negl Trop Dis. 2013. 10.1371/journal.pntd.0002400. 23991244 10.1371/journal.pntd.0002400 PMC3749975

[30] Zaman S, Nahar P, Macgregor H, Barker T, Bayisenge J, Callow C, et al. Severely stigmatised skin neglected tropical diseases: a protocol for social science engagement. Trans R Soc Trop Med Hyg. 2020;114(12):1013–20. 33324991 10.1093/trstmh/traa141 PMC7738656

[31] GREENE J, BASILICO MT, KIM H, FARMER P, Farmer P, Kim JY et al. Colonial Medicine and Its Legacies. In: Reimagining Global Health [Internet]. 1st ed. University of California Press; 2013. pp. 33–73. (An Introduction). Available from: http://www.jstor.org/stable/10.1525/j.ctt46n4b2.7

[32] Baer HA, Medical Pluralism BT-. Encyclopedia of Medical Anthropology: Health and Illness in the World’s Cultures Volume I: Topics Volume II: Cultures. In: Ember CR, Ember M, editors. Boston, MA: Springer US; 2004. pp. 109–16. Available from: 10.1007/0-387-29905-X_12

[33] Stahnisch FW, Biomedical, Dominance. Twentieth Century, and the Establishment of Biomedical Experts BT - Palgrave Encyclopedia of the Health Humanities. In: Crawford P, Kadetz P, editors. Cham: Springer International Publishing; 2020. pp. 1–9. Available from: 10.1007/978-3-030-26825-1_55-1

[34] Braun V, Clarke V. Qualitative Research in Psychology Using thematic analysis in psychology Using thematic analysis in psychology. Qual Res Psychol [Internet]. 2006;3(2):77–101. Available from: http://www.tandfonline.com/action/journalInformation?journalCode=uqrp20%5Cnhttp://www.tandfonline.com/action/journalInformation?journalCode=uqrp20

[35] Rose G. Visual Metodhelogis. 4th ed. Sage Publications Ltd.; 2016. p. 1–23.

[36] Kleinman A. Patients and Healers in the Context of Culture: An Exploration of the Borderland between Anthropology, Medicine, and Psychiatry [Internet]. 1st ed. Vol. 5. University of California Press; 1980. Available from: http://www.jstor.org/stable/jj.2711689

[37] Musyimi CW, Mutiso VN, Nandoya ES, Ndetei DM. Forming a joint dialogue among faith healers, traditional healers and formal health workers in mental health in a Kenyan setting: towards common grounds. J Ethnobiol Ethnomed. 2016;12(1):1–8. 10.1186/s13002-015-0075-6. 26742992 10.1186/s13002-015-0075-6 PMC4705751

[38] Kottai R, Ranganathan S. S. Task-Shifting in Community Mental Health in Kerala: Tensions and Ruptures. Med Anthropol Cross Cult Stud Heal Illn [Internet]. 2020;39(6):538–52. Available from: 10.1080/01459740.2020.1722122 10.1080/01459740.2020.1722122 32101053

[39] Nieto-Sanchez C, Hatley DM, Grijalva MJ, Grietens KP, Bates BR. Communication in Neglected Tropical Diseases’ elimination: A scoping review and call for action. PLoS Negl Trop Dis [Internet]. 2022;16(10):1–22. Available from: 10.1371/journal.pntd.0009774 10.1371/journal.pntd.0009774 PMC9595560 36228006

[40] Nasr Elsheikh M, Ackley C, Hall V, Zaman S. Because people here are ignorant’: the failure of a community intervention to prevent Mycetoma in Sudan. NIHR Open Res. 2023;3(January):2.

[41] Varas-díaz N, Grove K, Rodríguez SR. Decolonial visual resistance as a public health strategy in post-María. Puerto Rico. 2022;8(1):1–33. 10.1386/jvpc_00011_1 PMC9194790 35707717

[42] Brase GL, Richmond J. The white-coat effect: physician attire and perceived authority, friendliness, and attractiveness. J Appl Soc Psychol. 2004;34(12):2469–81.

[43] Loucks EB, Berkman LF, Gruenewald TL, Seeman TE. Relation of social integration to inflammatory marker concentrations in men and women 70 to 79 years. Am J Cardiol. 2006;97(7):1010–6. 16563907 10.1016/j.amjcard.2005.10.043

[44] Uchino BN. Social support and health: a review of physiological processes potentially underlying links to disease outcomes. J Behav Med. 2006;29(4):377–87. 16758315 10.1007/s10865-006-9056-5

[45] Fahal AH, Hotez PJ. Mycetoma. A global medical and socio-economic dilemma. PLoS Negl Trop Dis. 2017;11(4):e0005509. 10.1371/journal.pntd.0005509. 28426654 10.1371/journal.pntd.0005509 PMC5398501

[46] Emmanuel P, Dumre SP, John S, Karbwang J, Hirayama K, Mycetoma. A clinical dilemma in resource limited settings [Internet]. Vol. 17, Annals of Clinical Microbiology and Antimicrobials. 2018 [cited 2018 Dec 5]. p. 35. Available from: https://ann-clinmicrob.biomedcentral.com/articles/10.1186/s12941-018-0287-4 10.1186/s12941-018-0287-4 PMC6085652 30097030

[47] Amster EJ. Medicine and society:the past, present and future of race and colonialism in medicine. CMAJ. 2022;194(20):E708–10. 35609910 10.1503/cmaj.212103 PMC9188792

[48] Haynes E, Walker R, Mitchell AG, Katzenellenbogen J, Antoine HD, Bessarab D. Social Science & Medicine Decolonizing Indigenous health: Generating a productive dialogue to eliminate Rheumatic Heart Disease in Australia. Soc Sci Med [Internet]. 2021;277:113829. Available from: 10.1016/j.socscimed.2021.113829 10.1016/j.socscimed.2021.113829 33895707

[49] Mendizábal-Cabrera R, Pérez I, Becerril Montekio V, Pérez F, Durán E, Trueba ML. Cutaneous leishmaniasis control in Alta Verapaz (northern Guatemala): evaluating current efforts through stakeholders’ experiences. Infect Dis Poverty [Internet]. 2021;10(1):1–12. Available from: 10.1186/s40249-021-00842-3 10.1186/s40249-021-00842-3 PMC8106169 33962699

[50] Pérez, I., Durán, E., Pérez, F., Trueba, M., & Renata Mendizábal-Cabrera. (2020). Strengthening Cutaneous Leishmaniasis control in Guatemala: policy recommendations. PAHO Policy Briefing, June 2020. https://www.paho.org/en/documents/policy-brief-strengthening-cutaneous-leishmaniasis-control-guatemala-policy.

[51] Njamnshi AK, Angwafor SA, Tabah EN, Jallon P, Muna WFT. General public knowledge, attitudes, and practices with respect to epilepsy in the Batibo Health District, Cameroon. Epilepsy Behav. 2009;14(1):83–8. 10.1016/j.yebeh.2008.09.012. 18845276 10.1016/j.yebeh.2008.09.012

[52] Preux PM, Tiemagni F, Fodzo L, Kandem P, Ngouafong P, Ndonko F, et al. Antiepileptic therapies in the Mifi Province in Cameroon. Epilepsia. 2000;41(4):432–9. 10756409 10.1111/j.1528-1157.2000.tb00185.x

[53] Fahal A, Mahgoub ES, Hassan AMEL, Abdel-Rahman ME. Mycetoma in the Sudan: An Update from the Mycetoma Research Centre, University of Khartoum, Sudan. PLoS Negl Trop Dis [Internet]. 2015 Mar [cited 2017 Oct 4];9(3):e0003679. Available from: http://www.ncbi.nlm.nih.gov/pubmed/25816316 10.1371/journal.pntd.0003679 PMC4376889 25816316

